# Assessment of Digital Image Correlation Effectiveness and Quality in Determination of Surface Strains of Hybrid Steel/Composite Structures

**DOI:** 10.3390/ma17143561

**Published:** 2024-07-18

**Authors:** Paweł J. Romanowicz, Bogdan Szybiński, Mateusz Wygoda

**Affiliations:** 1 Department of Machine Design and Composite Structures, Faculty of Mechanical Engineering, Cracow University of Technology, ul. Warszawska 24, 31-155 Cracow, Poland; bogdan.szybinski@pk.edu.pl; 2 Department of Product Technology and Ecology, College of Management and Quality Sciences, Cracow University of Economics, 31-510 Cracow, Poland; mateusz.wygoda@uek.krakow.pl

**Keywords:** digital image correlation, strain analysis, adhesive joints, metal/composite structures, finite element method, notches, experimental tests

## Abstract

The application of the digital image correlation (DIC) contactless method has extended the possibilities of reliable assessment of structure strain fields and deformations throughout the last years. However, certain weak points in the analyses using the DIC method still exist. The fluctuations of the results caused by different factors as well as certain deficiencies in the evaluation of DIC accuracy in applications for hybrid steel/composite structures with adhesive joints are one of them. In the proposed paper, the assessment of DIC accuracy based on the range of strain fluctuation is proposed. This relies on the use of a polynomial approximation imposed on the results obtained from the DIC method. Such a proposal has been used for a certain correction of the DIC solution and has been verified by the introduction of different error measures. The evaluation of DIC possibilities and accuracy are presented on the examples of the static tensile tests of adhesively bonded steel/composite joints with three different adhesives applied. The obtained results clearly show that in a non-disturbed area, very good agreement between approximated DIC and FEM results is achieved. The relative average errors in an area, determined by comparison of DIC and FEM strains, are below 15%. It is also observed that the use of approximated strains by polynomial function leads to a more accurate solution with respect to FEM results. It is concluded that DIC can be successfully applied for the analyses of hybrid steel/adhesive/composite samples, such as determination of strain fields, non-contact visual detection of faults of manufacturing and their development and influence on the whole structure behavior during the strength tests, including the elastic response of materials.

## 1. Introduction

The available computer-aided engineering (CAE) software, like finite element analysis, boundary element method, finite volume or finite difference methods, multibody dynamics analysis, etc., provides in-depth support during the designing process of various mechanical structures [1]. Contemporary CAE software enables tracing the whole cycle of designing, starting from the simulation of different manufacturing processes through intermediate design stages up to the final new structure or product elaboration. Such a process is made in virtual space and is justified by economic reasons. Additionally, the application of CAE software can also be applied in the optimization or regeneration processes of structures or machine elements. However, analyses of practical problems by means of CAE methods require certain smaller or larger model simplifications, which may even lead to questionable results. Because of this, further experimental verification, like strain or deformation determination static or fatigue strength verification, of the real structure is still necessary and strongly recommended in many branches of industry [2,3]. 

Strains and deformations of structures can be assessed by several well-recognized experimental methods, like strain gauge application or photoelasticity [4,5]. The first one belongs to the contact techniques of deformation measuring [6,7,8], while the second one is the contactless technique. Such methods are very often used in Structural Health Monitoring (SHM) systems to monitor changes in the geometrical or material properties of engineering structures. In SHM, measuring systems used are piezoelectric sensors [9], fiber optic sensors [10,11], acousto-ultrasonic fiber sensors [12], and fiber-optic Fabry-Perot interferometers [13], among others [14]. These techniques have become promising alternative methods of deformation assessment in recent years. The last group can be divided into interferometric and non-interferometric methods. These methods are generally based on the comparison of the measured physical variables identifying the surface of the investigated structure before and after deformation. The difference between interferometric and non-interferometric methods relies on the application of the different light sources and different physical parameters to measure. In the case of interferometric methods, the coherent source of light must be used, and deformations are estimated on the basis of the phase change of the scattered light reflected from an investigated surface. Whereas the non-interferometric methods rely on the analysis of gray intensity changes of the tested object surface caused by deformation. Here, less strict requirements for a source light are demanded. Due to the possibilities of full-field strain (deformation) measurements, such non-contact methods have become more and more popular in practical applications. One of the most common non-interferometric techniques is the digital image correlation (DIC). This method was proposed by Peters and Ranson in 1982 [15]. The first practical applications of the DIC method were made for rigid body dynamics problems [16] and full-field in-plane deformations [17]. The detailed reviews of the two-dimensional DIC for in-plane displacement and strain measurements were discussed in [18,19], whereas a critical assessment of the DIC method’s capabilities and its future tasks is discussed by Pan [20]. Other non-interferometric methods suitable for the measurements of displacements and strain are grid methods [21]. On the other hand, the most popular interferometric methods are Moiré interferometry [22], speckle, and holography methods [23]. 

Recently, DIC has been one of the most popular optical techniques for full-field surface strain measurements [24]. The DIC technique theory for the determination of in-plane strains was developed by Peters et al. in 1985 [25]. In this method, the full-field surface deformation and strains are calculated on the basis of the identification and tracking of a corresponding speckle pattern registered before and after deformation. Generally, such a pattern is made by white (background) and black aerosol speckles on the object’s surface. The size and distribution of black speckles should be set with respect to the scale of the investigated surface. It is recommended to use random or various unique shapes of the pattern. DIC systems are proposed for 2D measurements of flat surfaces in which one camera is required in measurement [26] and 3D measurements of flat and curved objects in which at least two cameras correlated together are necessary to use [27]. Present-day cameras and algorithms provide sufficient accuracy of displacements (even 1 μm) and strain measurements. However, the obtained results can be affected by technical factors such as the number of cameras [28], the presence of out-of-plane translation, displacement, or rotation [28,29], image (or camera) noise [24], camera lens distortion [29], bad quality of a speckle pattern [30], or working environment (i.e., lighting) [29]. The significant influence on the accuracy of DIC calculations also has software algorithm properties such as sub-pixel intensity interpolation scheme and optimization algorithm [24,29,31], subset shape function, subset size, etc. [31,32,33].

In the recent literature, verifications of DIC were made by means of a comparison of DIC results with FEM solutions. Kosmann et al. [34] investigated 2D-DIC accuracy on the example of single-lap joints with thick aluminum adherend shear test specimens bonded by epoxy film adhesive. Srilakshmi and Ramji [35] tested the adhesively bonded composite patch on a cracked aluminum plate, and they used DIC for the determination of shear strain in the adhesive layer. The DIC and FEM comparison of strains in the adhesive layer of the double-sided patch-repaired composite bonded joint was also studied by Kashfuddoja and Ramji [36]. The above-mentioned studies confirmed that the DIC technique allows for inspection and strain determination along the adhesive joint. Seon et al. [37] applied DIC and the FEM for the determination of the interlaminar tensile strength and elastic properties of composite materials. They tested flat samples with circular holes and used the relative absolute strain error and the weighted root mean square error in order to compare FEM and DIC results. The root mean square error was also used for the assessment of DIC accuracy by Wang and Pan [33]. They investigated the influence of different DIC settings (subset size and DIC algorithm) on the error range. Peng et al. [38] compared DIC and FEM results for flat metal samples with an inclined notch. Lemmen et al. [39] studied two problems—the static crack extension test of fiber metal laminates (the strain fields ahead of a crack tip notch) and bonded joints with thick adherents. Based on the comparison of DIC and FEM results, they concluded that DIC makes it possible to visualize and evaluate phenomena that may not be visible or registered in FE analyses. Aidi and Case [40] applied DIC and FEM methods for a quasi-static tension test of composite samples with circular holes. They obtained qualitatively similar strain contours in both methods; however, the maximal strains around the notch in FEM were more than two times larger than the maximal DIC strains. The comparison of DIC and FEM plastic strains in steel plates with different notches (circular, rectangular, and triangular) subjected to static tension was performed by Romanowicz et al. [41]. The application of the DIC method in such a case was able to designate the growth of the plastic strains and zones with large strains in which further damage may initiate. All the above-mentioned studies confirm the good compatibility of DIC and FEM analyses and the clear benefits of the application of the DIC method during the experimental tests.

Despite the number of studies related to the assessment of DIC quality and accuracy, there are still certain deficiencies in the assessment of DIC quality and accuracy. These mainly refer to the application of DIC analyses for steel and hybrid structures working only in elastic regimes. In such structures, the strain level is significantly lower than in the above-mentioned structures, for which comparisons of DIC and FEM are performed. It should be noted that the above analyses are generally applied to phenomena in which quite large strains or deformations occur. These issues include analyses of composite samples with cracks or notches, adhesive layers applied, aluminum samples, etc. Such structures have smaller stiffness moduli or they contain large strain (stress) concentrations. This significantly increases the accuracy of DIC analyses in comparison with analyses of steel structures or structures without stress (strain) concentrations. This has significant meaning in the experimental studies of hybrid steel/composite adhesively bonded structures in which it is necessary to determine or monitor the strain (or deformation) field not only in the adhesive layer but also on the surfaces of steel cores and composite overlays. In the case of hybrid structures, such as steel/composite and concrete/composite with adhesively bonded joints, there are certain phenomena that require special analyses. These phenomena are related to the studies of interfacial stress transfer by the adhesively bonded joints, the stress concentrations at the fillet adhesive welds [42,43], failure mechanisms under fatigue loading conditions and reduction of the stress concentration around the notch in the core material [44], non-uniformity of stress and strain in the joint [45], and complex failure modes [46,47]. Another scientific challenge is the analysis of the stress transfer between two different materials with different stiffness, which can result in strain fluctuations and stress concentrations. Recent studies revealed wide possibilities for the application of the DIC method in both static [42,43] and fatigue [44] experimental analyses of hybrid steel/composite adhesively bonded and concrete/composites hybrid structures [46] joints in the determination of strain field. However, the main question, which is also the main aim of this study, concerns the quality and accuracy of the obtained DIC results. In the above-mentioned studies, DIC was used for monitoring failure mechanisms under different loading conditions. In recent publications, the measured strains by means of the DIC method were, in general, only qualitatively compared with FEM solutions, and mainly for materials with a low elastic modulus or working in an elastic-plastic regime. There is no detailed qualitative and quantitative verification of DIC technique possibilities and accuracy in application to measurements of full-field elastic strains in hybrid structures working in an elastic regime. In particular, there are no such analyses for steel structures in an elastic state. This missing gap is studied and discussed in detail in the paper. It has been revealed that it is possible to measure elastic strains in steel cores with high accuracy. This fact has an important meaning in experimental full-field strain measurements and significantly expands the possibilities of using the DIC technique in practical applications.

The quality and accuracy, as well as the possibilities of the DIC method with the use of a high-resolution camera, are assessed in the paper on the example of three notched steel samples reinforced by composite overlays. Such samples are made with different adhesives. The accuracy is evaluated on the basis of the fluctuation of DIC strains as well as on the basis of the comparison of DIC and FEM solutions. The performed analyses included the determination of strain fields at the steel core, composite overlays, and adhesive chamfered endings. 

The paper consists of five sections. The introduction and the literature review are given in Section 1. The descriptions of the applied methodology, including a brief overview of the basics of DIC, materials and samples, measurement systems, FEM models, and DIC systems, are given in Section 2. The results of the analyses, including measurements of adhesive thickness (Section 3.1), validation of the FEM model (Section 3.2), a full-scale DIC and FEM surface strain study (Section 3.3), comparison of the DIC and FEM results (Section 3.4), and accuracy and error analyses (Section 3.5), are presented in Section 3. A discussion of the presented study is provided in Section 4. The conclusions are given in Section 5.

## 2. Methodology

### 2.1. 2D DIC Background

**Figure 1:**
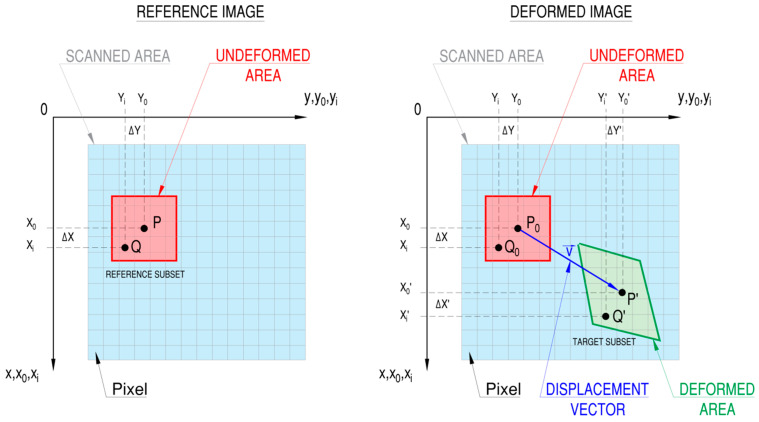
Illustration of the principle of DIC method operation with a reference subset (**left-hand side**) and an evaluated target subset (**right-hand side**).

The main principle of the DIC method is tracking and calculating the motion of points located on the investigated surface by comparing high-resolution images made for different loading conditions. The reference image, which should be made for the non-loaded structure, is divided by the virtual grid presented in Figure 1. 

The full-field deformation is achieved by the calculation of displacements on the investigated surface at points designated by this virtual grid. It is made by matching or tracking the same points (subsets) between images of the un-deformed reference structure and the deformed one. Generally, calculations are made for square reference subsets (with center point P) with a specified number of pixels. Such a technique allows for the matching of particular subsets through the analysis of the variations gray levels. This method is more accurate than the comparison of the movement of single points. This is due to the fact that the use of division in subsets ensures wider variations in gray levels in a particular subset, which distinguishes each subset from the other ones [18]. This makes it possible for more precise and unique identification of subsets before and after deformations. 

Comparison of the location of particular subsets—before and after deformation—allows for the calculation of the displacement vector at a selected point in a subset (generally the subset center). The displacement of the other points (sub-pixels) in the same subset is calculated by the utilization of sub-pixel interpolation schemes and displacement mapping functions as follows [18,48]:

(1) $$\left\{\begin{array}{c} x_{i}^{\prime }=x_{i}+\xi (x_{i},y_{j})\\ y_{j}^{\prime }=y_{j}+\eta (x_{i},y_{j}) \end{array}\right.,\;i,j=-M\ldots M,$$

where *ξ* and *η* are the shape functions, and the subset size is (2*M* + 1) × (2*M* + 1).

The shape functions should be selected in such a way as to enable accurate determination of the deformation form. In general, the shape functions can be described as follows [49]:

(2) $$\left\{\begin{array}{c} \xi \left(x_{i},y_{j}\right)=\xi_{0}+\xi_{1}x+\xi_{2}y+\xi_{3}x^{2}+\xi_{4}y^{2}+\xi_{5}xy\\ \eta \left(x_{i},y_{j}\right)=\eta_{0}+\eta_{1}x+\eta_{2}y+\eta_{3}x^{2}+\eta_{4}y^{2}+\eta_{5}xy \end{array}\right..\;\;$$

In the practical application, the zero-order *(ξ_1_, ξ_2_, ξ_3_, ξ_4_, ξ_5_* = 0 and *η_1_, η_2_, η_3_, η_4_, η_5_* = 0; allow for determination of only translation), the first-order (*ξ_3_, ξ_4_, ξ_5_* = 0 and *η_3_, η_4_, η_5_* = 0; allow for determination of translation and rotation as well as normal and shear strains), and the second-order shape functions (all *ξ* ≠ 0 and *η* ≠ 0; then the determination of more complex non-linear deformation is allowed) are used.

In the simplest case, in which only displacements occur (without shear, rotation, etc.), the shape functions are equal to the corresponding values of the displacement vector (*ξ* = *u* and *η* = *v*). In general, the more complex first-order or second-order shape functions are used [18]. 

The matching procedure (correlation) of the subset location on the particular images is made by evaluating the similarity degree of the gray-scale values between un-deformed and deformed subsets with the use of the correlation criteria. This correlation procedure is performed in two steps. In the first one, the approximation of the speckle pattern in all subsets is carried out with the use of the interpolation functions *f*(*x*_i_,*y*_j_) for undeformed and *g*(*x*_i_′,*y*_j_′) for deformed structures. One of the commonly used interpolation functions is bi-cubic spline interpolation [48], defined as follows:

(3) $$f\left(x_{i},y_{j}\right)=\sum_{m=0}^{3}\sum_{n=0}^{3}a_{mn}x^{m}y^{n}\;$$

where *a_mn_* are interpolation coefficients. In the second step, the matching of each corresponding subset of un-deformed and deformed images is made with the use of correlation criterion. In this step, the shape of each subset in a deformed image is sought, and the interpolation function takes a value close to the gray-scale value of the corresponding subset in the reference (un-deformed) image. 

Basically, correlation criteria can be divided into two groups (Table 1): cross-correlation criteria (CC) and sum-squared difference the correlation criteria (SSD). It is observed that the basic and simplest CC and SSD criteria are sensitive to disturbances in the illumination of the investigated surface. This can lead to large errors during the matching procedure and improper determination of the strain field. The normalized criteria (NCC and NSSD) reveal insensitivity to the linear scale in illumination lighting by the application of the parameters $\bar{f}$ and $\bar{g}$ (defined in Table 1). However, both criteria are still vulnerable to an offset from the light source. These normalized criteria are commonly used in commercial and research DIC software. Zero-normalized criteria show the least sensitivity for noise, lighting disturbances, and lightning offset. The value of correlation coefficient *C* is within the range [0, ∞), and the aim is to minimize its value (minimization of differences between functions *f* and *g*). 

The minimum value of the correlation criterion can be found by applying the Newton-Raphson method [48] as follows:

(4) $$\nabla \nabla C\left(P_{0}\right)\left(P-P_{0}\right)=-\nabla C\left(P_{0}\right)\;$$

where $\nabla C\left(P_{0}\right)$ is the gradient of *C*, $\nabla \nabla C\left(P_{0}\right)$ is the Hessian matrix (the second-order gradient of *C*), ***P*** is the next iterative approximate function, and ***P_0_*** is an initial guess of the solution.

In the next step, the deformation gradient tensor can be calculated, and finally, the full-field surface deformation and strains can be achieved.

**Table 1:** Correlation criteria commonly used in digital image correlation analyses.

Type	Name	Definition	
Cross-correlation criteria	Cross-correlation	$C_{CC}=\sum_{i=-M}^{M}\sum_{j=-M}^{M}\left[f\left(x_{i},y_{j}\right)g(x_{i}^{\prime },y_{j}^{\prime })\right]$	(5)
	Normalized cross-correlation	$C_{NCC}=\sum_{i=-M}^{M}\sum_{j=-M}^{M}\left[\frac{f\left(x_{i},y_{j}\right)g(x_{i}^{\prime },y_{j}^{\prime })}{\bar{f}\bar{g}}\right]$	(6)
		$\bar{f}=\sqrt{\sum_{i=-M}^{M}\sum_{j=-M}^{M}{\left[f(x_{i},y_{j})\right]}^{2}},\;\;\;\bar{g}=\sqrt{\sum_{i=-M}^{M}\sum_{j=-M}^{M}{\left[g(x_{i}^{\prime },y_{i}^{\prime })\right]}^{2}}$	(7)
	Zero-normalized cross-correlation	$C_{ZNCC}=\sum_{i=-M}^{M}\sum_{j=-M}^{M}\left\{\frac{\left[f\left(x_{i},y_{j}\right)-f_{m}\right]\times [g\left(x_{i}^{\prime },y_{j}^{\prime }\right)-g_{m}]}{∆f∆g}\right\}$	(8)
		$f_{m}=\frac{1}{{\left(2M+1\right)}^{2}}\sum_{i=-M}^{M}\sum_{j=-M}^{M}f\left(x_{i},y_{j}\right),\;$	(9)
		${\;g}_{m}=\frac{1}{{\left(2M+1\right)}^{2}}\sum_{i=-M}^{M}\sum_{j=-M}^{M}g\left(x_{i}^{\prime },y_{j}^{\prime }\right),$	(10)
		$∆f=\sqrt{\sum_{i=-M}^{M}\sum_{j=-M}^{M}{\left[f\left(x_{i},y_{j}\right)-f_{m}\right]}^{2},\;}∆g=\sqrt{\sum_{i=-M}^{M}\sum_{j=-M}^{M}{\left[g\left(x_{i}^{\prime },y_{j}^{\prime }\right)-g_{m}\right]}^{2}}$	(11)
Sum-squared difference correlation criteria	Sum of squared differences	$C_{SSD}=\sum_{i=-M}^{M}\sum_{j=-M}^{M}{\left[f\left(x_{i},y_{j}\right)-g(x_{i}^{\prime },y_{j}^{\prime })\right]}^{2}$	(12)
	Normalized sum of squared differences	$C_{NSSD}=\sum_{i=-M}^{M}\sum_{j=-M}^{M}{\left[\frac{f(x_{i},y_{j})}{\bar{f}}-\frac{g(x_{i}^{\prime },y_{j}^{\prime })}{\bar{g}}\right]}^{2}$	(13)
	Zero-normalized sum of squared differences	$C_{ZNSSD}=\sum_{i=-M}^{M}\sum_{j=-M}^{M}{\left[\frac{f\left(x_{i},y_{j}\right)-f_{m}}{∆f}-\frac{g\left(x_{i}^{\prime },y_{j}^{\prime }\right)-g_{m}}{∆g}\right]}^{2}$	(14)

### 2.2. Materials and Samples

The static tensile tests are made for notched samples reinforced by means of composite overlays. As a basic material for the core, the steel S355J2+N is used. Such a steel is widely used in metallic structures due to its good weldability and relatively high resistance. Such steel is low alloy steel with approximately 0.15% weight inclusion of carbon and manganese, which is the main and dominant alloy addition (approximately 1.33% of weight). The estimated (in tension tests) value of the yield stress for that steel is equal to 427 MPa, while the ultimate stress reaches 528 MPa at minimum. The chemical composition and basic mechanical properties of the investigated steel are summarized in Table 2. For overlays, the S&P C-Laminate 150/2000 (S&P Reinforcement Poland, Malbork, Poland) is used, which exhibits relatively high tensile resistance, particularly for tension directions parallel to the orientation of the composite fibers (Table 3). Three different structural adhesives are used in the performed tests. The first one, recommended by the manufacturer of the S&P C-Laminate, is the S&P Resin 220 Epoxy Adhesive (S&P Reinforcement Poland, Malbork, Poland) (sample 1_S&P220), which has a relatively high modulus of elasticity (*E* = 7000 MPa) and exhibits grainy structure after hardening. Such a kind of adhesive belongs to the group of adhesives showing a rather brittle form of failure. The next adhesive is the 3M Scotch-Weld DP6310NS (3M Poland Sp. z o.o., Kajetany, Poland) (sample 2_DP6310NS), which is a typical polyurethane structural adhesive recommended for metal/metal, metal/composite, and composite/composite connections of structural and layered elements. It is much softer than S&P Resin 220 (here, the elastic modulus *E* = 590 MPa), and its elongation at break is several times higher than for the former glue. The third adhesive is LOCTITE^®^ HY4080GY (Henkel Polska Sp. z o.o., Warszawa, Poland) (sample 3_HY4080GY). This adhesive is a two-component cyanoacrylate/acrylic adhesive with rather low modulus (*E* = 355 MPa), high viscosity, and large elongation at break. Due to its properties, it is recommended for connections of various materials. The last two adhesives have a ductile nature of destruction. The basic mechanical properties of the applied laminate and the three adhesives chosen for tests are summarized in Table 3. The materials are selected on the basis of the experimental fatigue tests described in [44], in which it is observed that the application of the composite overlays significantly increases the fatigue life of notched steel structures. It is also revealed that the main factor determining fatigue life is the type of adhesive. The average increase in fatigue life is as follows: HY4080GY—890%, DP6310NS—307%, and S&P Resin 220—97%. For a more general assessment of the DIC method in the paper, the measurements are performed for all adhesives. The reasons for application and limitations of using composite materials and adhesives are discussed in Ref. [44]. A detailed description of the procedure for sample preparation is also described in Ref. [44]. 

**Figure 2:**
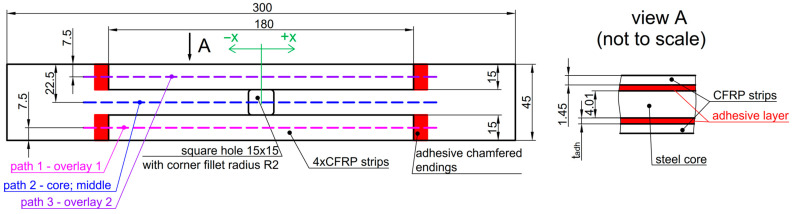
Geometry of tested samples, location of paths 1–3, and definition of axis *x*.

**Figure 3:**
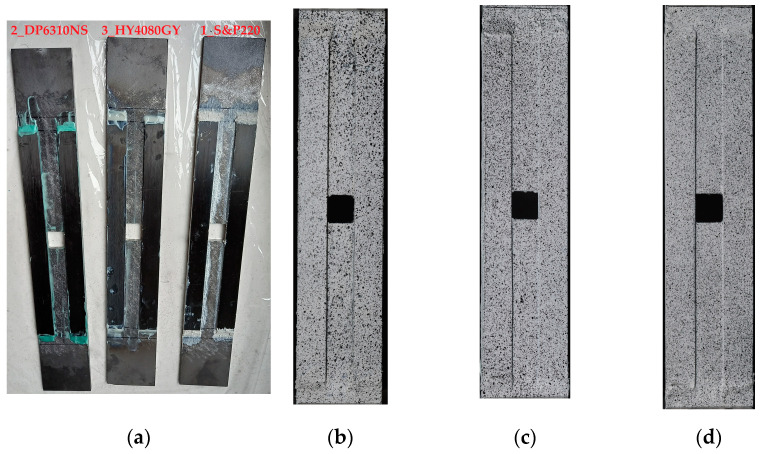
Photographs of tested samples: (**a**) after gluing before painting and with speckle patterns applied, (**b**) 1_S&P220, (**c**) 2_DP6310NS, and (**d**) 3_HY4080GY.

In all tested samples, the notch of the same form is applied. In the center of the sample, a square hole with rounded corners is cut. Such a kind of notch is commonly applied (besides circular openings) to various thin-walled profiled structural beams, folded plates, and other structural notched elements. In the tests, bare steel plates with the geometry shown in Figure 2 are investigated. Only one value for the rounded corner radius is used as follows: *R* = 2 mm, which can be regarded as a sharp notch. The reinforcing overlays have the form of four long stripes placed symmetrically along the sample length and the hole on the top and bottom surfaces of the steel core. The images of the tested samples are shown in Figure 3. Here, samples with glued overlays before they were covered with a speckle pattern (Figure 3a) and samples coated with speckle patterns (Figure 3b–d) are presented. The paths 1–3, along which strains are studied, are shown in Figure 2. The experimental static tensile tests are carried out with the use of a MTS Landmark 370 testing machine (MTS Systems, Eden Prairie, MN, USA) with a tensile speed of 0.5 mm/min.

### 2.3. DIC System

**Figure 4:**
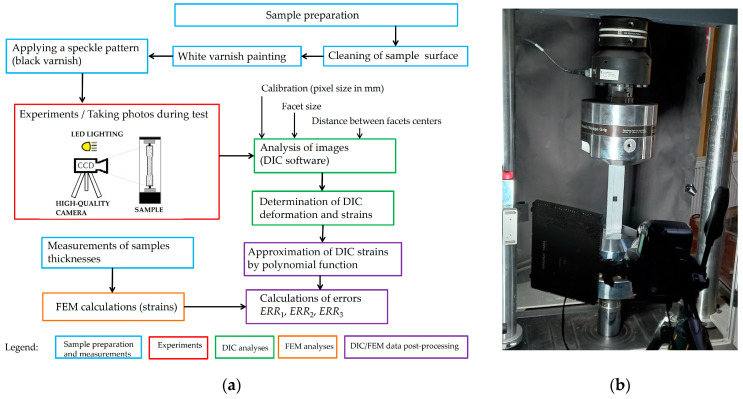
DIC measurement and analysis system: (**a**) flowchart with applied methodology and (**b**) photo of the measurement system.

The DIC system (Figure 4) used in the presented study consists of the digital camera (Nikon D-90 (Nikon Imaging Japan Inc., Tokyo, Japan)) equipped with an AF-S NIKKOR 50 mm f/1.8 G lens (Nikon Imaging Japan Inc., Tokyo, Japan), the external light source (LED), and the GOM Correlate Software 2018 Hotfix 1 [50]. The camera was additionally equipped with an external trigger system. In each tested case, the images were taken for unloaded samples (the reference images) and for the sequence of the chosen loads (tension) applied to the samples. The resolution of the image was 4288 × 2848 pixels. In such a case, one pixel corresponded to 0.052 mm in length on the surface of the investigated object. The main settings of DIC analyses were facet size and the distance between the center points of the facets. The main criterion for the selection of both above-given parameters was to reduce the fluctuations of the strain distribution, which are common for the DIC method. The final configuration was chosen on the basis of the preliminary analyses of the obtained results. Finally, due to the high resolution of the images, the size of the facets size was set to 150 pixels, and the distance between their centers was set to 50 pixels. After converting the facet size and distance into a unit length, they were equal to 8.1 mm and 2.7 mm, respectively.

### 2.4. FEM Model

The numerical calculations of the investigated sample are made by means of the Ansys program Campus version 2022R2 (ANSYS Inc., Canonsburg, PA, USA) [51], which is a well-established finite element method software. Taking into account the dimensions of the investigated object—small thickness in comparison to the length and width of the sample—two kinds of analysis can be applied to investigated samples. These are 2D and 3D approaches. In the case of the 2D modeling, the analysis can be made with shell elements, namely, SHELL181 elements, which are well suited for the analysis of laminated composite shells and plane panels. Such an approach—due to its definition—offers relatively fast result acquisition. Unfortunately, the existence of a relatively large zone of adhesive chamfered endings, which is located at the end of each overlay, strongly violates the results of simulations. So, the most suitable and effective approach relies on the application of 3D solid elements like SOLID185. Such a finite element (FE) is the eight-node brick finite element with 3 degrees of freedom at each node. It also has plasticity, large deflection, large strain, capabilities, and others, which are crucial for reliable result acquisition. In the investigated cases, the real non-linear true stress-true strain curve for the core (steel S355J2+N) is implemented (multi-linear elastic-plastic material model with kinematic hardening) [51].

**Figure 5:**
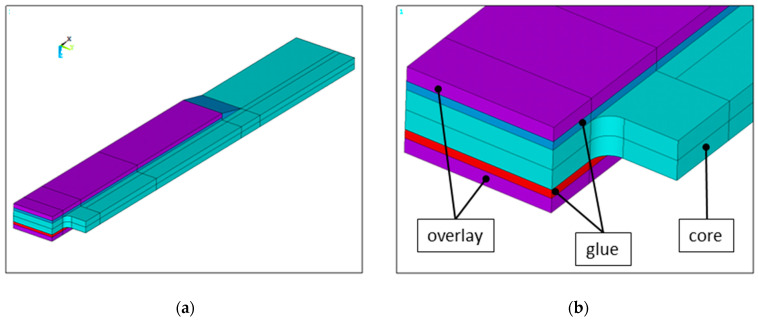
General view of the model: (**a**) quarter part of the investigated structure and (**b**) magnification of the zone with a rectangular hole.

Due to the sample symmetry of geometry and applied loads, only one-fourth of the full structure is modeled. The general idea behind the use of the FE model is presented in Figure 5.

**Figure 6:**
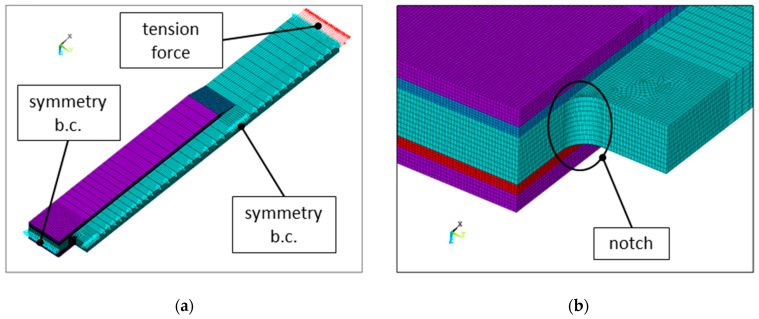
Finite element model with mesh and boundary conditions applied: (**a**) quarter part of the investigated structure and (**b**) magnification of the zone with a rectangular hole.

The main difficulty in model generation in FE analysis arises from two factors: the shape of the applied overlays and the presence of a notch in the corner of the metallic core located in the close vicinity of the overlay edge. The shape of the sample influences the volume and finite element mesh generation, which results in the presence of very small distorted (wedge-shaped) elements at the sharp corner of the notch, which influences the quality of the results (see Figure 6). In this figure, the coarse mesh is shown.

### 2.5. Proposed Methodology for Evaluation of DIC Accuracy

The accuracy of DIC analyses depends on many factors related to surface preparation and image registration, as well as DIC analysis settings. One of the key parameters is the subset size and distance between the centers of surrounding subsets (defined as “point distance”). The size of subsets is set with respect to a pattern (size and density of the pattern features) made on the tested surface. Each subset should contain at least three pattern features. Too small a subset size leads to higher displacement noise (fluctuations of results), whereas too large a subset size results in a worse determination of local effects (smoothing effect). The density of measurement points is determined by point distance. Decreasing its value results in increasing the density of measurement points; however, it increases computation time.

In order to evaluate the quality of DIC analysis, the three measures of “errors” are introduced. The first one is related to the amplitude of the fluctuations of the obtained DIC strains. In order to obtain high accuracy in DIC analyses, such fluctuations should be minimized. Because of this, the amplitude of the fluctuations is assumed to be the main measure of DIC accuracy. The fluctuation error *ERR*_1_ is calculated as the relative difference between the strain calculated from DIC ε_x*,DIC*_ and the approximated strain ε*_x,APPR_*, as follows:

(15) $${ERR}_{1}\left(\%\right)=\frac{\varepsilon_{x,DIC}-\varepsilon_{x,APPR}}{\varepsilon_{x,APPR}}\cdot 100\%$$

The fluctuation error strongly depends on the quality of the images (optical noise, source, and kind of light used, lens distortion, surface pattern, etc.) as well as on the settings of the DIC analysis (facet size, distance between facets, number of points, etc.). 

The second “error” measure is determined by a comparison of the approximated DIC $\varepsilon_{x,APPR}$ and calculated by FEM strains with the following formula:

(16) $${ERR}_{2}\left(\%\right)=\frac{\varepsilon_{x,APPR}-\varepsilon_{x,FEM}}{\varepsilon_{x,FEM}}\cdot 100\%$$

The third “error” measure is determined by comparison of the $\varepsilon_{x,DIC}$ designated directly by the software DIC and calculated by FEM strains as follows:

(17) $${ERR}_{3}\left(\%\right)=\frac{\varepsilon_{x,DIC}-\varepsilon_{x,FEM}}{\varepsilon_{x,FEM}}\cdot 100\%$$

The higher values of the above parameters ${ERR}_{2}$ and ${ERR}_{3}$ should be regarded as higher differences between DIC and FEM solutions. However, both measures can describe some errors that occurred during the analyses or other phenomena that have not been included in the FEM analyses (such as differences in the thickness of adhesive layers, etc.). 

## 3. Results

### 3.1. Measurements of Real Adhesive Thickness 

The thickness measurements were made with the use of a precision micrometer (Würth, Adolf Würth GmbH & Co. KG, Künzelsau-Gaisbach, Germany) with a measuring range of 0–25 mm, a scale value of 0.01 mm, and measuring spindle increments of 0.5 mm. The particular thicknesses of the steel cores and overlays were measured before sample preparations and were equal to *t_core_* = 4.01 mm (standard deviation SD: 0.007 mm) and *t_ovls_* = 1.45 mm (SD: 0.008 mm), respectively.

**Figure 7:**
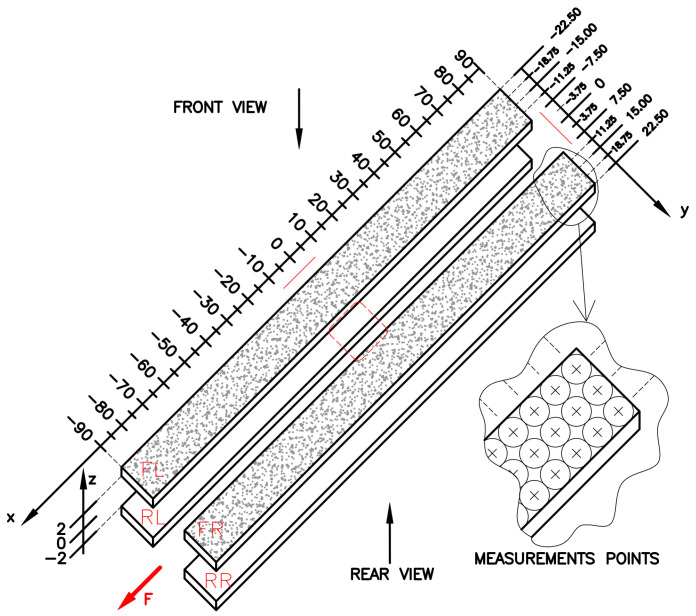
Scheme of sample thickness measurements.

The thickness measurements of the prepared samples (with overlays) were made along the entire length of the overlays. The first measurements were taken at a distance of 5 mm from the short side of the overlay. Further measurements were made every 10 mm along the longer side of the overlay. In each measuring distance (along the *x*-axis), measurements were made at three equidistantly located points (along the *y*-axis) with a pitch equal to 3.75 mm. This led to the measurements being 54 points for each overlay. The general view of the measured procedure is presented in Figure 7.

The average, constant thicknesses (required in FEM analyses) of the adhesive layers were calculated as the difference between the thicknesses of the prepared samples (*t_samp_*), steel core (*t_core_*), and overlays (*t_ovl_*) using the following formula:

(18) $$t_{adh,avg}=0.5\left(t_{samp}-t_{core}-2t_{ovl}\right)$$

They are given in Table 4. 

**Figure 8:**
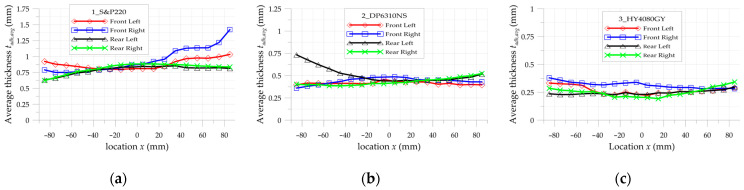
Distribution of average thickness of adhesive layer in sample: (**a**) 1_S&P220, (**b**) 2_DP6310NS, and (**c**) 3_HY4080GY.

In order to evaluate the particular detailed adhesive thicknesses, the additional thickness measurements were made after tests, after the debonding of the overlays from the steel core. Such measurements were made with the same procedure as is pointed out in Figure 7. Results of this study are presented in Figure 8a–c for 1_S&P220, 2_DP6310NS, and 3_HY4080GY samples, respectively. 

### 3.2. Validation of the FEM Model 

In order to properly choose the approximation level, the three meshes, namely, coarse, moderate, and dense, are studied. Their choice is determined by several factors. First of all, at least three or more divisions (elements) are applied across the width of the thinnest part (here glue), and the divisions across the width of the core and the overlays are fitted to have an almost uniform height of all elements across the whole width. The second factor concerns the mesh shape at the notch area, particularly the part where the arc of the notch starts to extend outwards from the line defined by the longitudinal edge out of the overlay. In this area, it is difficult to generate finite elements in hexahedral shape, and mapped meshing is not fully provided. In the close vicinity of the sharp corner, the degenerated hexahedral—wedge elements—have to be applied. To estimate the adequacy of the finite element approximation, three different mesh densities are proposed and studied, and the following error measure proposed in the form given below is controlled:

(19) $$∆\sigma \%=\frac{\left(\sigma_{eqv}^{max}-\sigma_{bound}\right)}{\sigma_{bound}}\cdot 100\;\%$$

**Figure 9:**
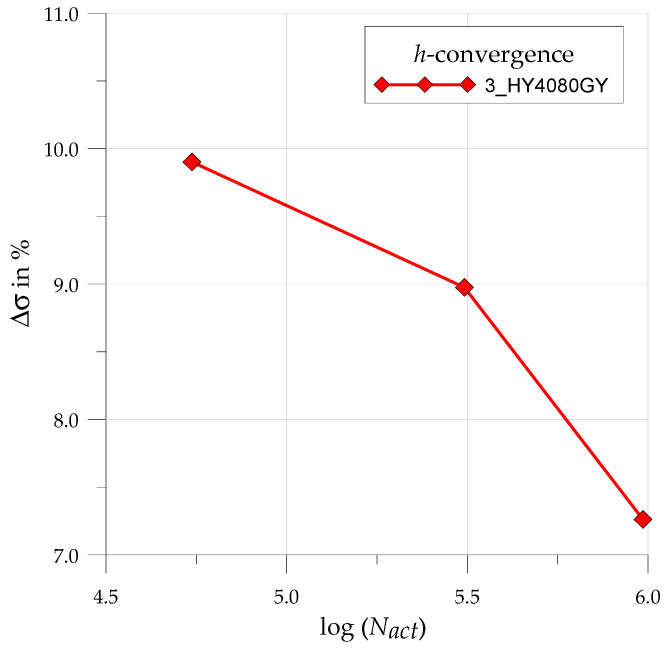
Exemplary results of the convergence study for the 3_HY4080GY sample.

Here, $\sigma_{eqv}^{max}$ is the maximum equivalent stress obtained in the notch area, while $\sigma_{bound}$ stands for the bounded maximum stress estimated by the software for the applied mesh. The results of that *h*-convergence study for Loctite HY4080GY adhesive are shown in Figure 9. Here is the distribution of ∆σ% with respect to the number of applied active degrees of freedom $N_{act}$. These degrees of freedom map the number of applied elements and mesh density. 

It is clearly seen that the properly chosen denser mesh provides better quality results. The final choice of the mesh is determined by the compromise between the value of the ∆σ% error, calculation time, and the maximum admissible size of the numerical task, which can be proceeded with in the accessible version of the Ansys software (ANSYS Inc., Canonsburg, PA, USA). In the final, densest mesh, the length of the rounded part (see Figure 6b) is divided into 22 segments. 

**Figure 10:**
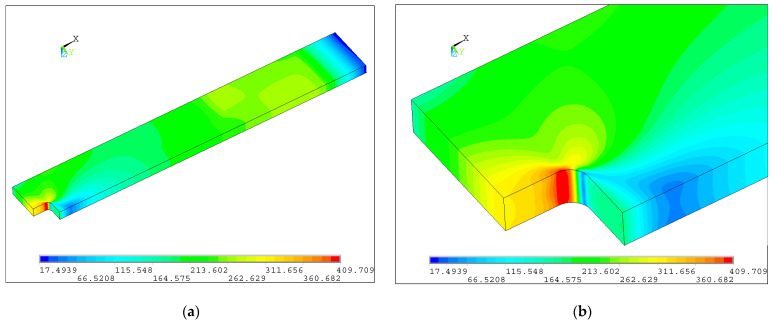
Distribution of equivalent von Mises stress (3_HY4080GY): (**a**) quarter part of the investigated structure and (**b**) magnification of the zone with a rectangular hole.

In the picture below (Figure 10), the distribution of the equivalent stress in the notch of the core part is shown (Loctite HY4080GY adhesive). These results are obtained for the finest, densest mesh, with approximately 1 million equations to solve for the one-fourth symmetric part of the full sample.

This mesh configuration is applied in the analysis of all three studied adhesives, namely: S&P Resin 220 (1_S&P220), 3M Scotch-Weld DP6310NS (2_DP6310NS), and Loctite HY4080GY (3_HY4080GY). The only change that is encountered in these samples concerns the set, constant width value of the adhesive, which changes from 0.27 mm (Loctite HY4080GY) through 0.62 mm (3M Scotch-Weld DP6310NS) to 0.86 mm (S&P Resin 220).

### 3.3. Full-Scale Surface Strain Study

**Figure 11:**
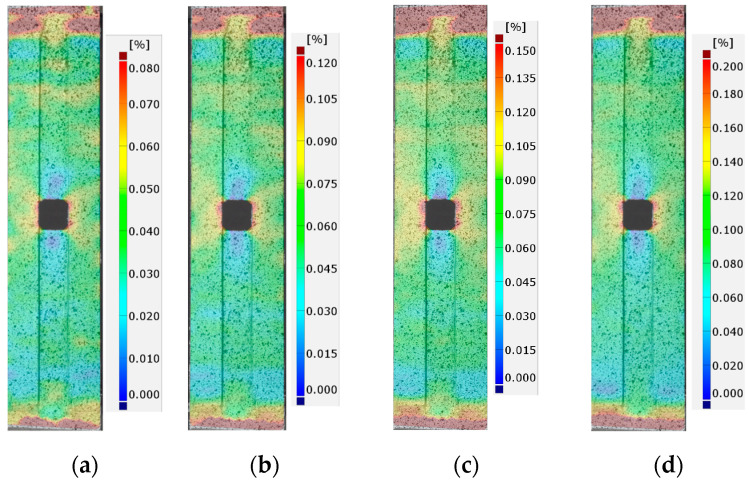
Distribution of total mechanical surface strains $\varepsilon_{x}$ (DIC) for sample 1_S&P220 and tensile force *F* equal to: (**a**) 20 kN, (**b**) 30 kN, (**c**) 40 kN, and (**d**) 47.5 kN.

**Figure 12:**
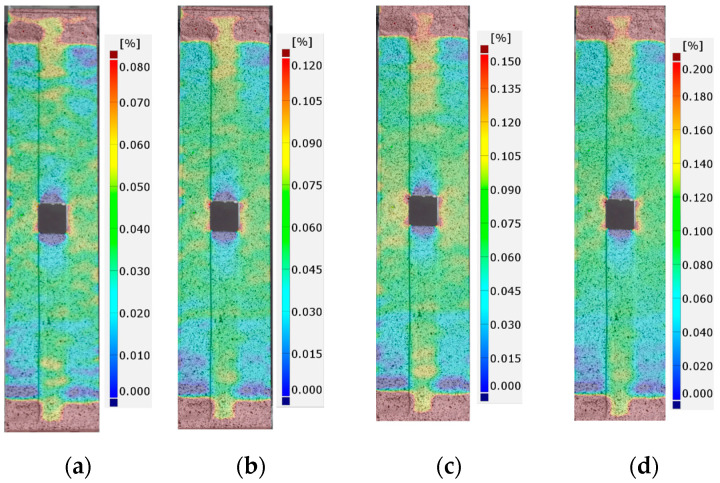
Distribution of total mechanical surface strains $\varepsilon_{x}$ (DIC) for sample 2_DP6310NS and tensile force *F* equal to: (**a**) 20 kN, (**b**) 30 kN, (**c**) 40 kN, and (**d**) 47.5 kN.

**Figure 13:**
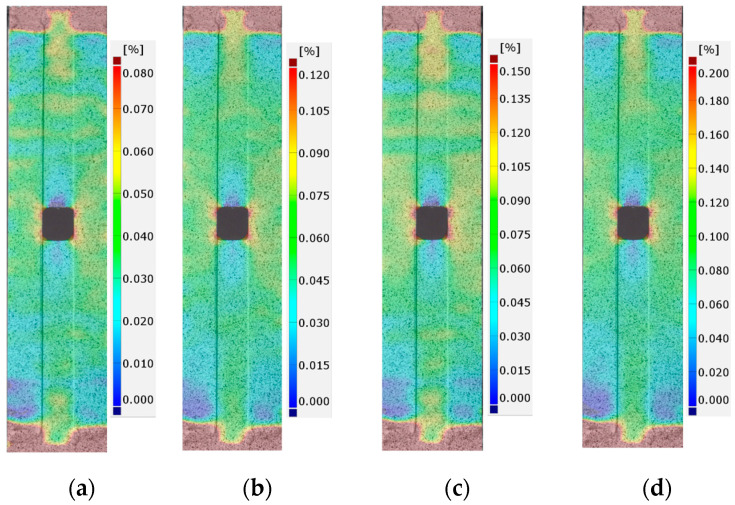
Distribution of total mechanical surface strains $\varepsilon_{x}$ (DIC) for sample 3_HY4080GY and tensile force *F* equal to: (**a**) 20 kN, (**b**) 30 kN, (**c**) 40 kN, and (**d**) 47.5 kN.

The detailed FEM and DIC analyses and comparisons were made for the total mechanical strains $\varepsilon_{x}$ measured along the tension direction. During the static tensile tests, the images were made for a few force levels: 0 kN (reference image), 10 kN, 20 kN, 30 kN, 40 kN, 45 kN, and 47.5 kN (the maximal applied tensile force). In each case, in order to make an image, a test was stopped at the specific load, for the time required to take the photo. The growths and distributions of surface strains calculated by DIC software (GOM Correlate) for tested samples with adhesives S&P 220 Resin, DP6310NS, and HY4080 are presented in Figure 11, Figure 12 and Figure 13, respectively. The results are shown for tensile forces of 20, 30, 40, and 47.5 kN. Strains that exceed the assumed maximal and minimal levels at the legends are marked as burgundy and navy blue colors, respectively.

In all cases, the significant strain concentrations caused by the presence of the notch are visible in the vicinity of the rounded corners of the rectangular hole. It is worth mentioning that such strain concentrations are observed on the external surface of the applied CFRP overlays. The next areas where large strain concentrations are also observed are the adhesive chamfered endings. However, such results are disturbed due to the following:

- change of the materials (stiff CFRP overlays/soft adhesive/stiff steel core).
- inclination of the adhesive chamfered endings with respect to the CFRP overlays and steel core.
- the rough and folded surface of the adhesive chamfered endings.

Because of this, interpretation of the results on both endings of the samples should be made with care. 

**Figure 14:**
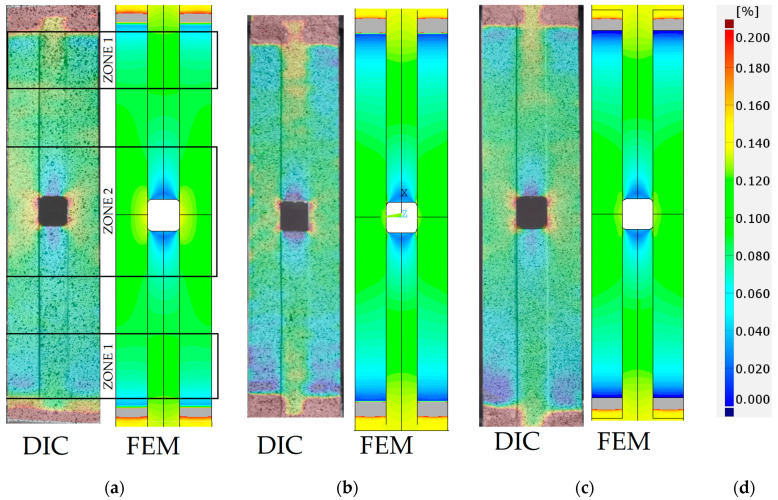
Comparison of DIC and FEM results (total mechanical strain $\varepsilon_{x}$) for maximal static load *F* = 47.5 kN for strengthened specimens with applied adhesive: (**a**) S&P Resin 220, (**b**) DP6310NS, (**c**) HY4080GY, and (**d**) strain legend.

The comparison of DIC and FEM results (Figure 14) of the strain calculations is made for the maximal applied static tensile force equal to 47.5 kN. In all cases, the same scale for strains is used. The scale applied in Figure 14 is set in such a way that it provides a legible and diversified distribution of deformations on the largest possible area of the tested samples. It should be noted that due to the small stiffness of the adhesives (in comparison with steel core and CFRP overlays), high deformations occurred at the spew fillets at the ends of the overlays. Such strains are significantly higher than the maximal value of the applied scale (ε = 0.2), and due to that, they are marked as burgundy and gray in the DIC and FEM results, respectively. Three different adhesive bonds are compared in this study: S&P Resin 220 (Figure 14a), DP6310NS (Figure 14b), and HY4080GY (Figure 14c). 

In all cases, quantitatively and qualitatively good agreement between DIC and FEM results is observed. In particular, several characteristic features can be distinguished. The first one, which is observed at the ends of the overlays (zones 1), is related to the mechanism of tension forces transferring from the core to the overlays. When moving from the end of the overlays to the center of the sample, the growth of the strains on the overlay surface and the decrease in the strains in the steel core are observed along a certain length (zone 1).

The second one is the distinct differentiation of the strain distributions between CFRP overlays and steel core at the central part of the sample (zone 2). In the steel core, there are visible relatively low strain zones below and above the horizontal edge of the hole. What is more, the DIC analysis shows the occurrence of small compressive strains in such areas. Similar effects, which may even lead to local buckling, are also observed in References [52,53] for thin samples with holes subjected to tension loads. The strains on the overlay surfaces are almost constant for the whole zone 2. Some differences are observed in the surroundings of the open hole, where strain concentrations on CFRP overlaps are visible in DIC results for all adhesives, while in the case of FEM analysis, such an effect appears only for S&P Resin 220. This is related to the differences in the stiffness of the adhesives applied in FEM calculations. The stiffnesses of HY4080GY and DP6310NS are significantly lower than S&P Resin 220, which significantly influenced the distribution of strains on CFRP overlays.

### 3.4. Comparison of DIC and FEM Results

**Figure 15:**
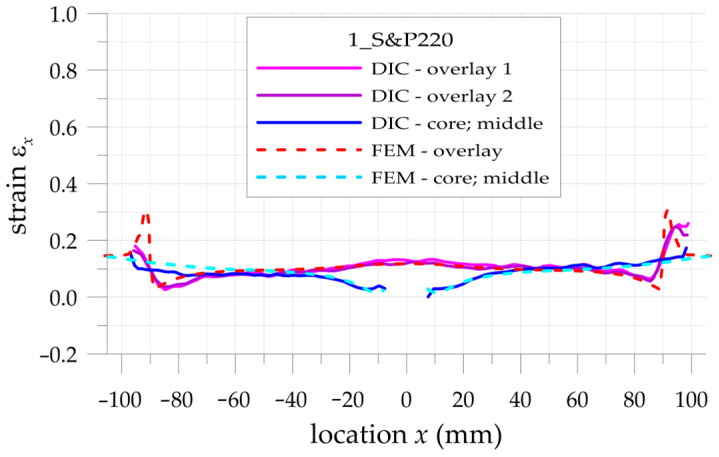
Distributions of calculated (FEM) and measured (DIC) total mechanical strains on the surface of the sample with S&P Resin 220 adhesive.

**Figure 16:**
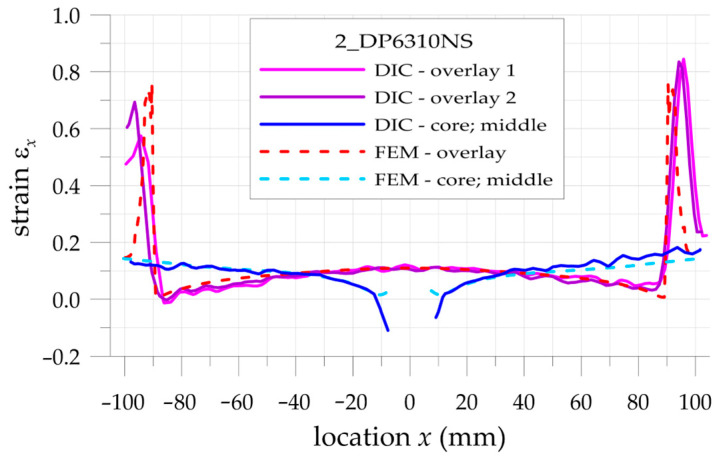
Distributions of calculated (FEM) and measured (DIC) total mechanical strains on the surface of the sample with DP6310NS adhesive.

**Figure 17:**
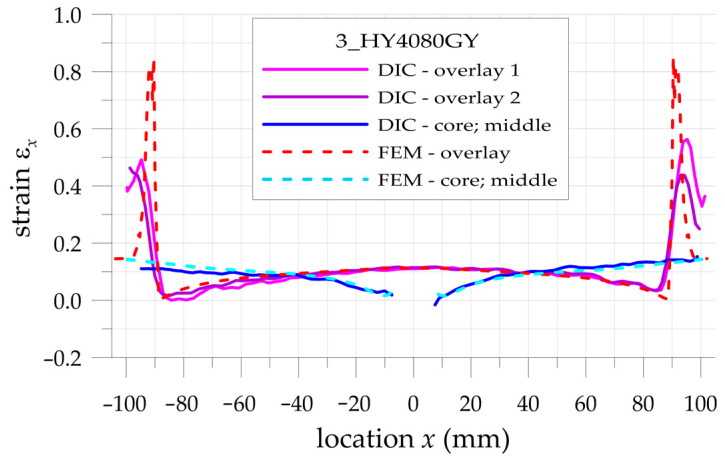
Distributions of calculated (FEM) and measured (DIC) total mechanical strains on the surface of the sample with HY4080GY adhesive.

The more detailed comparison and analyses of the accuracy and possibilities of DIC measurements are carried out for the three paths presented in Figure 2. Two paths 1 and 3 are defined on the outer surfaces of both overlays in their half-width. Path 2 is defined on the surface of the steel core at the central axis of the sample. Such analyses are performed for all samples with S&P Resin 220 (Figure 15), DP6310NS (Figure 16), and HY4080GY (Figure 17) adhesives. The results are presented for the maximal tensile load *F* = 47.5 kN. The obtained experimental results are compared with FEM solutions for particular paths and adhesives. 

Such a preliminary comparison revealed the high compatibility of DIC and FEM results in the central part of the sample ($x\in \left(-50,\;50\right)\;mm)$. However, in such a range, some differences in strains in the core and in the surrounding area of the hole edge can be observed. Slightly larger differences can be observed in the zones x<−50 mm and x>50 mm. The largest differences occurred at the adhesive chamfered endings (see Table 5). However, this effect can be explained by using the catalog Young’s moduli of tested adhesives in FEM analyses. Such catalog values are generally given as the smallest ones, and this fact contributed to the discrepancy in the obtained results. The largest difference is observed for HY4080GY, which means that the applied Young modulus of this adhesive is highly underestimated in the performed FEM analysis. 

The main problem in DIC analysis is that the obtained distributions of vertical strains ε_x_ exhibit certain fluctuations. The range of disturbances depends on different factors such as the quality of speckled patterns, the properties of DIC analysis (i.e., size of the facets and distance between facets centers, etc.), lighting stability, photo noise, distortion, etc. In order to eliminate such fluctuations, smoothing procedures were proposed and performed for all obtained DIC results. For that purpose, the following approximation function is used:

(20) $$\varepsilon_{x,APPR}=b_{0}+b_{1}x+b_{2}x^{2}+b_{3}x^{3}$$

where *b_i_* are the approximation coefficients.

Obviously, the strains calculated by DIC at the boundaries of the analyzed surfaces as well as in the areas where different materials are joined (particularly with different stiffnesses) are distorted due to the averaging of the results.

**Figure 18:**
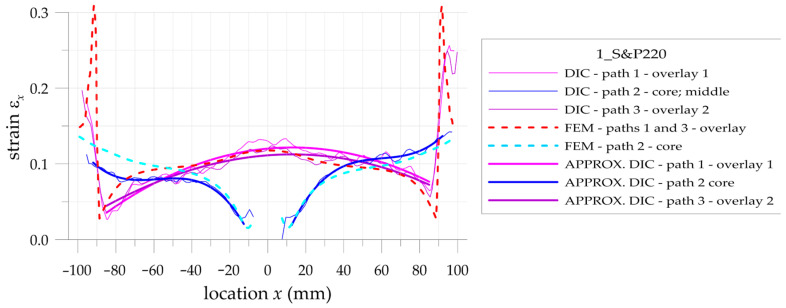
Comparison of calculated (FEM) and measured (DIC) and approximated (DIC) total mechanical strains on the surface of the sample with S&P Resin 220 adhesive.

**Figure 19:**
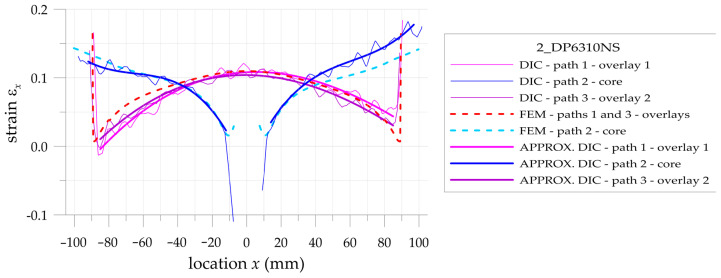
Comparison of calculated (FEM) and measured (DIC) and approximated (DIC) total mechanical strains on the surface of the sample with DP6310NS adhesive.

**Figure 20:**
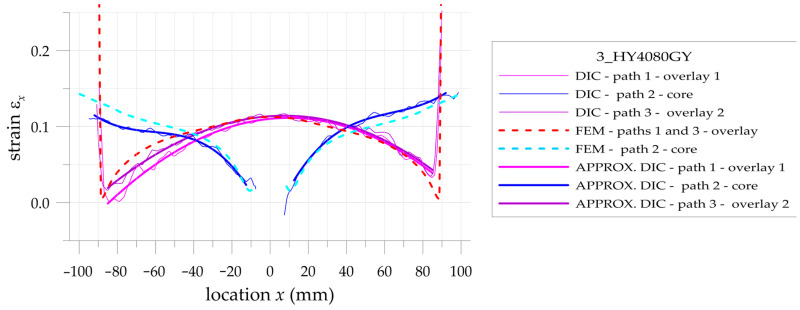
Comparison of calculated (FEM) and measured (DIC) and approximated (DIC) total mechanical strains on the surface of the sample with HY4080GY adhesive.

In the case of analyzed samples and for the assumed properties of DIC analysis, the maximal range of the distorted results at the boundaries does not exceed 5 mm. Then, the abovementioned approximation is limited to the undistorted areas. The obtained strains directly calculated by DIC (thin lines), strains calculated by the use of FEM (dashed lines), and approximated strains from DIC (thick lines) are shown in Figure 18, Figure 19 and Figure 20 for samples with S&P Resin 220, DP6310NS, and HY4080GY adhesives, respectively. The proposed approximation allowed for the reduction of the typical fluctuations of strains observed in DIC analysis. 

More detailed comparisons of strains calculated by DIC and FEM are shown in Table 6 and Table 7. For that purpose, certain locations (*x =* −80, −60, −40, −20, 0, 20, 40, 60, 80) are chosen. In the case of DIC strains, the direct strains calculated by GOM Correlate and the approximated strain according to Formulae (20) are depicted. The comparison of the results for overlays is given in Table 6 and includes strains for both front overlays (paths 1 and 3—Figure 2). A similar comparison made for the steel core is presented in Table 7. 

### 3.5. Accuracy and Error Analyses

The main limitation of the DIC method is the presence of a large error in strain determination near the edge of the selected area of the analysis (which can also be the edge of the investigated structure). In all investigated cases, the same properties of the DIC analyses are applied (see Section 2.3). The calculations of the errors ${ERR}_{1}$, ${ERR}_{2}$, and ${ERR}_{3}$ are performed for the areas of the samples, excluding the surrounding edges of the hole or ends of overlays where huge errors occurred. All presented results are for tensile load *F* = 47.5 kN. Based on the obtained results (analysis error rate), the value of this offset is assumed to be equal to 5 mm (facet size—8.1 mm, distance between facet centers—2.7 mm). 

The particular errors are determined on three paths defined on the outer surface of each sample along the tensile direction in the middle part of them as follows:

- both overlays (paths 1 and 3—Figure 2).
- the steel core (path 2—Figure 2).

**Figure 21:**
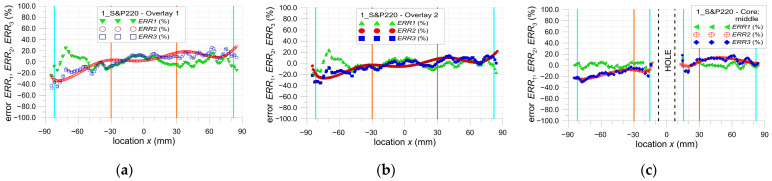
Distribution of error ${ERR}_{1}$, ${ERR}_{2}$, and ${ERR}_{3}$ parameters for sample 1_S&P220: (**a**) path 1—overlay 1, (**b**) path 3—overlay 2, and (**c**) path 2—core.

**Figure 22:**
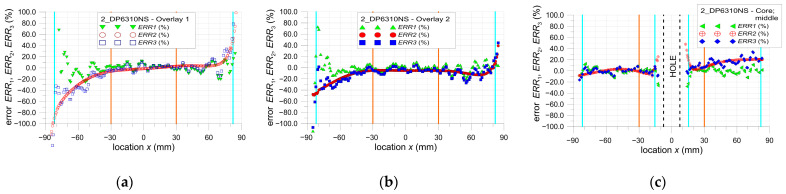
Distribution of error ${ERR}_{1}$, ${ERR}_{2}$ and ${ERR}_{3}$ parameters for sample 2_DP6310NS: (**a**) path 1—overlay 1, (**b**) path 3—overlay 2, and (**c**) path 2—core.

**Figure 23:**
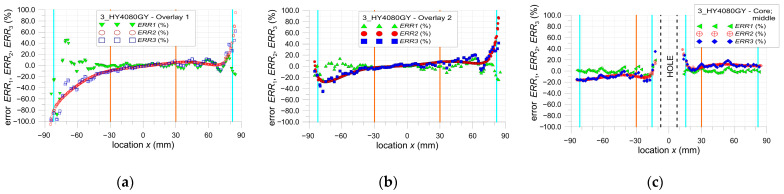
Distribution of error ${ERR}_{1}$, ${ERR}_{2}$ and ${ERR}_{3}$ parameters for sample 3_HY4080GY: (**a**) path 1—overlay 1, (**b**) path 3—overlay 2, and (**c**) path 2—core.

They are presented in Figure 21, Figure 22 and Figure 23. Additionally, certain vertical lines are added to all the plots mentioned. The dashed-black lines define the location of the square hole. The cyan lines define the internal edges of the facets located at the border of the investigated area. In the case of paths 1 and 2, these lines define the area within the range $x\in \left(-82,\;82\right)$, and in the case of path 3, within the range $x\in \left(-82,-15.5\right)\cup \left(15.5,\;82\right)$. Moreover, due to the quality of the images taken by the camera, the investigated area is divided into two parts—the center part $x\in \left(-30,\;30\right)$ and external zones $x\in \left(-85,-30\right)\cup \left(30,\;85\right)$. Such areas are separated by the orange lines. This division into two zones is justified by the fact that the images taken by the camera are disturbed by some errors. These are mainly optical radial distortion (barrel, pincushion, and mustache), chromatic aberration, and optical vignetting. Generally, the growth of the optical radial distortion increases when moving outside the center of the image. All images are made in such a way that the centers of the images are set at the center of the sample hole. Because of this, the highest accuracy of the surface mapping is obtained in the center part of the image $x\in \left(-30,\;30\right)$. In the external zones ($x\in \left(-85,-30\right)\cup \left(30,\;85\right)$), the images can be affected by the abovementioned optical errors. This also leads to a decrease in the DIC analysis accuracy in the external zones. 

Finally, the whole investigated area of the sample is divided into a few subregions. In such subareas, the average absolute error calculated from all points taken into consideration in DIC analyses is calculated. Additionally, the maximal absolute errors are also collected. The summary of the obtained results is given in Table 8 (for overlays) and Table 9 (for steel core). The smallest average absolute errors are observed in the central part of the investigated area—$x\in \left(-50,\;50\right)$ for all paths. In such a range, the values of these errors do not exceed 15%. In the remaining part, the observed errors reached larger values. Such phenomena refer mainly to the strains on overlay surfaces. This is caused by certain simplifications of the FEM model (constant thicknesses were assumed in the FEM model while real thicknesses were in fact variable—in Figure 8, the distributions of adhesive layer thicknesses can be observed) and larger distortions of the images. 

The largest errors are observed for the maximal absolute values of errors. However, such big values generally occurred at single or a few points located close to the chamfered adhesive endings or in the neighborhood of the hole.

## 4. Discussion

**Figure 24:**
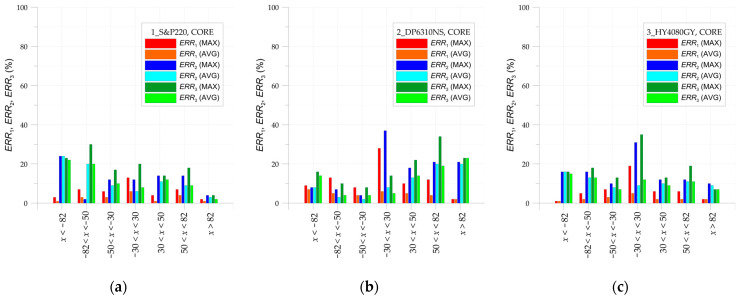
Distribution of errors ${ERR}_{1}$, ${ERR}_{2}$, and ${ERR}_{3}$ in steel core in sample: (**a**) 1_S&P220, (**b**) 2_DP6310NS, and (**c**) 3_HY4080GY.

**Figure 25:**
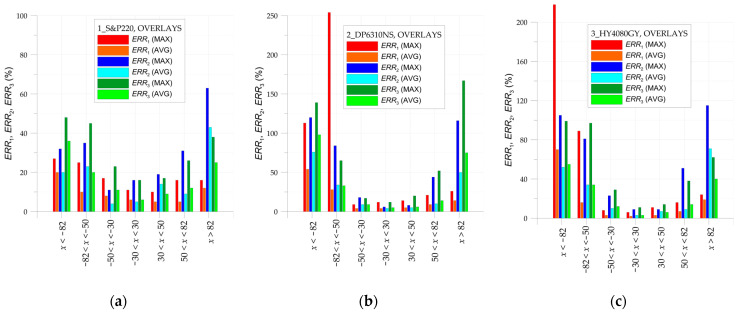
Distribution of errors ${ERR}_{1}$, ${ERR}_{2}$, and ${ERR}_{3}$ in overlays in sample: (**a**) 1_S&P220, (**b**) 2_DP6310NS, and (**c**) 3_HY4080GY.

The calculated values of the maximal absolute observed and average absolute errors in particular sub-regions are graphically summarized in Figure 24 (path 2—steel core) and Figure 25 (paths 1 and 3—overlays). It can be seen that the evaluated errors in the analyses of the strains at the steel core (Figure 24) are relatively stable and, except for singular cases, do not exceed 20% in the whole investigated range. The distributions of errors in overlays (Figure 25) are significantly different. In this case, in all investigated samples, the errors ${ERR}_{2}$ and ${ERR}_{3}$ (comparison of DIC and FEM strains) at both ends achieve large values. It should be noted that the average ${ERR}_{1}$, which depends on the fluctuation of DIC results, is generally significantly smaller than errors ${ERR}_{2}$ and ${ERR}_{3}$. This means that such large errors ${ERR}_{2}$ and ${ERR}_{3}$ are rather caused by phenomena that occurred during experimental tests and have not been included in the FE model. These are as follows:

- the mechanism of the load transfer from the steel core to the overlays, which may have a very complex nature.
- influence of the shape of adhesive-chamfered endings on the final results.
- variable adhesive thickness, which is not taken into account in the FE model.

It is worth noticing that the values of studied errors registered for path 2 (steel core) are significantly lower at the corresponding subregions for paths 1 and 3. This shows rather the deficiency of the FE model—particularly in the overlay zones—but not the inaccuracy of the DIC approach. The FE model used for comparison with DIC results is rather simple, so a better assessment of the FE results demands a more sophisticated approach to modeling, particularly in material modeling.

The final assessment of the accuracy of DIC direct solutions and DIC approximated solutions with FEM results is made with the use of the relative error defined as follows:

(21) $${∆}_{ERR}=\frac{{ERR}_{2}-{ERR}_{3}}{{ERR}_{3}}\cdot 100\%$$

Due to the fact that FEM models do not include the real distribution of adhesive thicknesses, the above final assessment is made in the restricted zone limited to $x\in \left(-50,50\right)$ length. The average and maximal errors *ERR*_2_ and *ERR*_3_ calculated with the use of obtained values are given in Table 10. The negative values of relative error ${∆}_{ERR}$ mean that more accurate results are obtained when using approximated strains in calculations. The positive values of ${∆}_{ERR}$ mean that more accurate results are obtained when using direct DIC strains. The obtained ${∆}_{ERR}$ revealed that more accurate results are obtained for strains calculated with the use of the introduced polynomial approximation. Taking into account the average values of errors *ERR*_2_ and *ERR*_3_ only in one case (sample 2_DP6310NS and core), the direct strains provide more compatible results with FEM than the approximated strains. In the whole investigated range $x\in \left(-50,50\right)$, the values of both average errors (*ERR*_2_ and *ERR*_3_) do not exceed 10%. A similar trend is observed for the maximal values of the errors; however, the largest ${∆}_{ERR}=68.2\%$ obtained for sample 2_DP6310NS and the core is caused by a few large values of approximated strain in the surroundings of the rectangular hole. In the whole investigated range $x\in \left(-50,50\right)$, the values of both maximal errors (*ERR*_2_ and *ERR*_3_) do not exceed 40%. However, it should be noted that the biggest error values generally occurred at a few points located close to the hole edge.

By summarizing the above results, it can be concluded that, for a properly conducted experiment, the accuracy of the DIC method is satisfactory. The experimental analysis can also be extended with the use of a thermography camera. Such instrumentation (DIC and thermography camera) may have significant meaning in the case of preliminary static and fatigue tests of new materials, which require a detailed analysis of the behavior of materials or assessment of the damage growth of structural parts during complex fatigue tests [54]. 

## 5. Conclusions

The performed study proved the high opportunities and accuracy of the DIC method in application to strain measurements and monitoring of the hybrid steel/composite structures. On the basis of the presented results, the following conclusions can be drawn:

- DIC can be successfully applied for the measurements of elastic strains in hybrid steel/adhesive/composite samples, including elastic strains in the steel core.
- The error of strains evaluated by means of the DIC method can be assessed by the analysis of the amplitudes of the strain fluctuations.
- The application of the third-level polynomial approximation function allows for the reduction of the fluctuation of strains, which results in a more smoother distribution of surface strains and leads to a more accurate solution with respect to FEM results.
- In the central parts of the samples ($x\in \left(-50,50\right)),$ the errors *ERR*_2_ and *ERR*_3_ do not exceed 30% (the maximal absolute errors) and 15% (the average absolute errors) for overlays, and 35% (the maximal absolute errors) and 14% (the average absolute errors) for steel core. For a narrowed analysis area ($x\in \left(-30,30\right))$ and overlays, the errors *ERR*_2_ and *ERR*_3_ are reduced to 16% (the maximal absolute errors) and 6% (the average absolute errors). The values of the above-issued errors are fully acceptable for the measurements of strains in engineering structures.
- The largest differences between DIC and FEM results are observed on the overlays in the vicinities of the rounded corners of the rectangular holes. The observed strain concentrations in all samples in DIC analyses at such points are not noticed in FEM analyses.

The main limitation of popular DIC software relies on the lack of determination of strains at the boundaries of the structures (edges). Further development of such a technique, including the above-mentioned limitation, seems to be the main future direction of the investigations. This will provide the possibilities of reliable analysis of the strain concentration not only inside the structures but also at the notches and boundaries. Summarizing, the application of DIC analysis allows for non-contact visual detection of faults in manufacturing and their development and influence on the whole structure’s behavior during the strength tests. It is revealed that it is possible to measure surface elastic strains in hybrid steel/composite structures with sufficiently high accuracy with the use of the 2D DIC technique. This allows the DIC method to be used to control and measure full-field strains and to verify numerical and theoretical approaches using full-scale experimental strain measurements. The obtained results also provide further opportunities to use the DIC method to assess the influence of various factors occurring in adhesive joints on their fatigue life.

## Figures and Tables

**Table 2:** Chemical composition and material properties of the material used for steel cores.

Chemical Composition (in % Weight)									Minimal Yield Stress	Ultimate Tensile Strength (UTS)
C	Mn	Si	Al	Cu	Cr	S	P	Fe		
0.15	1.33	0.13	0.04	0.02	0.02	0.04	0.01	Res.	427 MPa	528 MPa

**Table 3:** Material properties of composite laminates and adhesives.

Material	Type	*E*_1_ (GPa)	*E*_2_, *E*_3_ (GPa)	*G*_12_, *G*_23_ (GPa)	*G*_31_ (GPa)	ν_1_, ν_2_	ν_3_	UTS (MPa)
S&P C-Laminate 150/2000	CFRP	165	10	5	0.5	0.3	0.03	2800
S&P Resin 220	Adhesive	7	-	-	-	-	-	14
DP 6310 NS		0.59	-	-	-	-	-	18.6
HY 4080 GY		0.355	-	-	-	-	-	11.3

**Table 4:** Average adhesive thicknesses in investigated samples.

Designation	Adhesive	Average Adhesive Thickness $t_{adh,avg}$ (mm)
1_S&P220	S&P Resin 220	0.86 (SD: 0.14 mm)
2_DP6310NS	DP 6310 NS	0.62 (SD: 0.18 mm)
3_HY4080GY	HY 4080 GY	0.27 (SD: 0.06 mm)

**Table 5:** Maximal strains in adhesive chamfered endings.

Designation	Maximal Surface Strain $\varepsilon_{x}$ (%)			
	x≈−90 mm		x≈90 mm	
	DIC ^(1)^	FEM	DIC	FEM
1_S&P220	0.18/0.16	0.31	0.26/0.25	0.31
2_DP6310NS	0.57/0.69	0.76	0.84/0.83	0.76
3_HY4080GY	0.49/0.44	0.85	0.56/0.44	0.85

^(1)^ Strains are given for both overlays in the form overlay 1/overlay 2.

**Table 6:** Comparison of DIC and FEM strains at selected points of overlays.

Location *x* (mm)	Maximal Surface Strain $\varepsilon_{x}$ (%) ^(1)^								
	1_S&P220			2_DP6310NS			3_HY4080GY		
	DIC	DIC APPR.	FEM	DIC	DIC APPR.	FEM	DIC	DIC APPR.	FEM
−80	0.038/0.045	0.044/0.051	0.066	0.025/0.036	0.008/0.021	0.038	0.005/0.025	0.010/0.029	0.038
−60	0.079/0.080	0.073/0.074	0.093	0.048/0.056	0.052/0.059	0.074	0.044/0.068	0.050/0.063	0.078
−40	0.095/0.089	0.096/0.092	0.097	0.076/0.080	0.082/0.085	0.091	0.079/0.090	0.080/0.088	0.094
−20	0.110/0.104	0.111/0.104	0.109	0.101/0.092	0.100/0.100	0.105	0.100/0.104	0.100/0.106	0.107
0	0.129/0.118	0.120/0.111	0.118	0.115/0.107	0.108/0.104	0.110	0.110/0.114	0.110/0.114	0.112
20	0.117/0.105	0.121/0.112	0.109	0.108/0.100	0.106/0.099	0.105	0.106/0.112	0.110/1.112	0.107
40	0.112/0.110	0.115/0.107	0.097	0.087/0.080	0.095/0.86	0.091	0.100/0.097	0.099/0.101	0.094
60	0.113/0.101	0.102/0.095	0.093	0.079/0.065	0.077/0.065	0.074	0.086/0.093	0.079/0.082	0.078
80	0.075/0.077	0.082/0.078	0.066	0.048/0.041	0.051/0.038	0.038	0.055/0.048	0.048/0.052	0.038

^(1)^ Strains are given for both overlays in a form overlay 1/overlay 2.

**Table 7:** Comparison of DIC and FEM strains at selected points of the steel core.

Location *x* (mm)	Maximal Surface Strain $\varepsilon_{x}$ (%)								
	1_S&P220			2_DP6310NS			3_HY4080GY		
	DIC	DIC APPR.	FEM	DIC	DIC APPR.	FEM	DIC	DIC APPR.	FEM
−80	0.088	0.084	0.113	0.125	0.113	0.120	0.099	0.101	0.120
−60	0.083	0.079	0.096	0.102	0.105	0.103	0.093	0.092	0.103
−40	0.076	0.079	0.086	0.091	0.091	0.089	0.089	0.083	0.090
−20	0.045	0.047	0.052	0.054	0.052	0.054	0.045	0.048	0.054
20	0.047	0.052	0.052	0.059	0.057	0.054	0.055	0.058	0.054
40	0.098	0.097	0.086	0.102	0.102	0.089	0.101	0.100	0.090
60	0.112	0.108	0.096	0.116	0.124	0.103	0.114	0.116	0.103
80	0.112	0.116	0.113	0.141	0.145	0.120	0.134	0.132	0.120

**Table 8:** Maximal and average value of absolute observed error (%) in the middle parts of overlays (paths 1 and 3).

		Investigated Zone (mm)						
		x<−82	$x\in \left(-82,-50\right)$	$x\in \left(-50,-30\right)$	$x\in \left(-30,30\right)$	$x\in \left(30,50\right)$	$x\in \left(50,82\right)$	x>82
		Maximal Absolute Observed Error/Average Absolute Error (%)						
1_S&P220	*ERR*_1_	27/20	25/10	17/8	11/6	10/5	16/5	16/12
	*ERR*_2_	32/20	35/23	11/4	16/5	19/14	31/9	63/43
	*ERR*_3_	48/36	45/20	23/11	16/6	17/9	26/12	38/25
2_DP6310NS	*ERR*_1_	113/54 ^(1)^	254/28 ^(2)^	9/4	12/4	14/5	21/9	26/14
	*ERR*_2_	120/76	84/34	18/9	6/4	8/5	44/10 ^(3)^	116/50
	*ERR*_3_	139/98	65/33	17/9	12/5	20/6	52/14 ^(3)^	167/75
3_HY4080GY	*ERR*_1_	218/70 ^(4)^	89/16	8/3	6/2	11/3	16/7	24/19
	*ERR*_2_	105/52 ^(4)^	81/34	23/10	9/3	9/7	51/9	115/71
	*ERR*_3_	99/55	97/34	29/12	11/3	14/6	38/14	62/40

^(1)^ Results only for path 3 (approximated strains on path 1 were close to 0), ^(2)^ large values (above 75%) occurred in three points on path 1 and are caused by very small values of approximated strains (close to 0—see Figure 19), ^(3)^ large values occurred in one point at the end of the investigated range (x≈81 mm), ^(4)^ large values occurred in one point on path 1 and were caused by very small values of approximated strains (close to 0—see Figure 20).

**Table 9:** Maximal and average value of absolute observed error (%) in the middle part of the core (path 2).

		Investigated Zone (mm)						
		x<−82	$x\in \left(-82,-50\right)$	$x\in \left(-50,-30\right)$	$x\in \left(-30,30\right)$	$x\in \left(30,50\right)$	$x\in \left(50,82\right)$	x>82
		Maximal Absolute Observed Error/Average Absolute Error (%)						
1_S&P220	*ERR*_1_	3/1	7/3	6/3	13/6	4/1	7/4	2/1
	*ERR*_2_	24/24	25/20	12/9	12/6	14/11	14/9	4/3
	*ERR*_3_	23/22	30/20	17/10	20/8	14/12	18/9	4/2
2_DP6310NS	*ERR*_1_	9/7	13/5	8/4	28/6 ^(1)^	10/5	12/4	2/2
	*ERR*_2_	8/8	7/3	4/2	37/8 ^(1)^	18/13	21/20	21/20
	*ERR*_3_	16/14	10/4	8/4	14/5	22/14	34/19	23/23
3_HY4080GY	*ERR*_1_	1/1	5/2	7/3	19/5	6/2	6/2	2/2
	*ERR*_2_	16/16	16/13	10/8	31/9	12/10	12/11	10/9
	*ERR*_3_	16/15	18/13	13/7	35/12	13/9	19/11	7/7

^(1)^ Large values occurred at a few points close to the hole edge.

**Table 10:** Comparison of DIC direct solutions and DIC approximated solutions based on FEM results for range $x\in \left(-50,50\right)$.

		*ERR*_2_ (AVG) %	*ERR*_3_ (AVG) %	*ERR*_2_ (MAX) %	*ERR*_3_ (MAX) %	${∆}_{ERR}$ (AVG) %	${∆}_{ERR}$ (MAX) %
1_S&P220	Core	6.9	9.3	12	20	−25.8	−40.0
	Overlays	5.5	7.3	19	23	−24.7	−17.4
2_DP6310NS	Core	6.5	5.9	37	22	10.2	68.2
	Overlays	5.0	5.4	18	17	−7.4	5.9
3_HY4080GY	Core	8.8	9.9	39	35	−11.1	11.4
	Overlays	5.3	5.3	23	29	0.0	−20.7

## Data Availability

The raw data supporting the conclusions of this article will be made available by the authors on request.
